# A familial *SAMD9* variant present in pediatric myelodysplastic syndrome

**DOI:** 10.1101/mcs.a006256

**Published:** 2023-04

**Authors:** Mahvish Q. Rahim, April Rahrig, Kathleen Overholt, Erin Conboy, Magdalena Czader, Amanda June Saraf

**Affiliations:** 1 Pediatric Hematology Oncology, Department of Pediatrics, Indiana University School of Medicine, Indianapolis, Indiana, USA;; 2 Riley Hospital for Children at Indiana University Health, Indianapolis, Indiana, USA;; 3 Department of Medical and Molecular Genetics, Indiana University School of Medicine, Indianapolis, Indiana 46202, USA;; 4 Department of Pathology and Laboratory Medicine/Indiana University Healthy Pathology Laboratory, Indiana University School of Medicine, Indianapolis, Indiana 46202, USA

**Keywords:** bone marrow hypocellularity, multiple lineage myelodysplasia

## Abstract

Myelodysplastic syndrome (MDS) is a rare pediatric diagnosis characterized by ineffective hematopoiesis with potential to evolve into acute myelogenous leukemia (AML). In this report, we describe a unique case of a 17-yr-old female with an aggressive course of MDS with excess blasts who was found to have monosomy 7 and a *SAMD9* germline variant, which has not previously been associated with a MDS phenotype. This case of MDS was extremely rapidly progressing, showing resistance to chemotherapy and stem cell transplant, unfortunately resulting in patient death. It is imperative to further investigate this rare variant to aid in the future care of patients with this variant.

## INTRODUCTION

Myelodysplastic syndrome (MDS) is a rare diagnosis in children with incidence of one to four patients per million children per year and accounts for 5% of new malignant diagnoses in children <18 yr of age (Niemeyer and Baumann 2008). MDS is a syndrome of ineffective hematopoiesis often presenting with cytopenia and an increased risk for transformation to acute myeloid leukemia (AML) (Locatelli et al. 1995). A diagnosis of pediatric MDS is often difficult to make because of the heterogenous clinical presentation and rarity when compared to adult patients (Niemeyer and Baumann 2008). Stem cell transplant (SCT) is the only curative therapy for pediatric MDS but carries a high risk of relapse and treatment related mortality (Guinan et al. 1989; Smith et al. 2013).

Currently, the World Health Organization (WHO) classification, which utilizes both morphology and genetic changes (Arber et al. 2016; Zhang et al. 2022), is used for classification of MDS. MDS can morphologically be classified based on blast count. A blast count of <5% in the bone marrow (BM) or <2% in the peripheral blood (PB) falls under the pediatric categorization of childhood MDS with low blasts (MDS-LB). MDS with increased or excess blasts (MDS-IB or MDS-EB) is classified into two categories with type 1 having 5%–9% blasts in the BM or 2%–4% in the PB and type 2 having 10%–19% blasts in the BM or 5%–19% blasts in the PB or presence of Auer rods (Khoury et al. 2022). The WHO distinguishes MDS from AML with a bone marrow blast threshold of 20%, or any blast percentage with an AML-defining genetic change. MDS with excess blasts (previously referred to as RAEB) is very aggressive and the most likely to progress to AML if left untreated (Mohammad 2018).

Although the majority of adult MDS is sporadic, pediatric MDS is often associated with inherited bone marrow failure syndromes or more recently identified genetic predisposition syndromes including *RUNX1*, *ANKRD26*, *ETV6*, *GATA2*, and *SAMD9/SAMD9L.* Chromosomal losses occur in ∼30% of primary childhood MDS (Niemeyer and Baumann 2008). Monosomy 7 and monosomy 5 are the most common chromosomal abnormalities (Niemeyer and Baumann 2008). *GATA2*, *CEBPA*, *RUNX1*, *ANKRD26*, *ETV6*, and *DDX41* are genes that are known to be associated with heritable forms of MDS and AML (Pastor et al. 2018). Other somatic (or just variants) variants associated with MDS such as *DNMT3A*, *TET2*, and *ASXL1* are highly predictive for disease evolution to AML in the adult population (Platzbecker 2019). There are several unfavorable variants associated with MDS in the following genes: *TP53*, *ASXL1*, *RUNX1*, *EZH2*, and *ETV6*. Patients with unfavorable variants in these genes often require more intensive up-front clinical care for the patient (Platzbecker 2019).

Germline disease-causing variants in *SAMD9* genes are present in ∼20% of pediatric patients with MDS (Wong et al. 2018). *SAMD9* is located at 7q21, which commonly has deletions in myeloid neoplasms (Schwartz et al. 2017a). *SAMD9* variants have been found to result in DNA damage repair defects and eventually apoptosis in hematopoietic cells as a precursor to MDS (Thomas et al. 2021). *SAMD9* variants are associated with monosomy 7 myelodysplasia and leukemia (M7MLS2). Other variants associated with M7 and MLS2 are K676E or E1136Q, both of which impair cellular growth. K676E suppresses cell cycle progression and E1136Q decreases proliferation and cell cycle progression (Wong et al. 2018; Thomas et al. 2021). The SAMD9 proteins are poorly characterized but have been linked to growth factor signaling and antiviral properties and are involved in control of cell proliferation, acting as a tumor suppressor in some cancers (Davidsson et al. 2018; Wong et al. 2018). *SAMD9* pathogenic variants are commonly associated with MIRAGE syndrome, which consists of five out of the six of the following clinical features: myelodysplasia, infection, growth restriction, adrenal hypoplasia, genital phenotypes, and enteropathy (Wong et al. 2018). *SAMD9* variants are described in MDS; however, there is not much known about the many variants that this gene may harbor and their impact on a patient's clinical course.

Here, we report a unique case of a patient with an aggressive course of MDS with excess blasts who was found to have monosomy 7 and a rare *SAMD9* germline variant. This is published with verbal consent from patient's next of kin, her mother.

## RESULTS

### Case Report

Our patient is a previously healthy 17-yr-old Hispanic female who was initially evaluated by her primary care provider for migraines ∼1 mo after a COVID-19 infection. Laboratory evaluation revealed a mild macrocytic anemia, leukopenia, and hyperbilirubinemia. Repeat evaluation a few months later showed a white blood cell count (WBC) 2.8 k/cumm, hemoglobin (Hgb) 10.1 gm/dL, mean corpuscular volume (MCV) 108.5 fl, platelets 234 k/cumm, 1.9% atypical lymphocytes, absolute neutrophil count (ANC) 0.98 k/cumm. Her iron studies were within normal limits and metabolic profile showed an elevated total bilirubin of 2.7 md/dL. A hepatitis panel was negative and abdominal ultrasound showed no significant findings.

**Figure 1.**
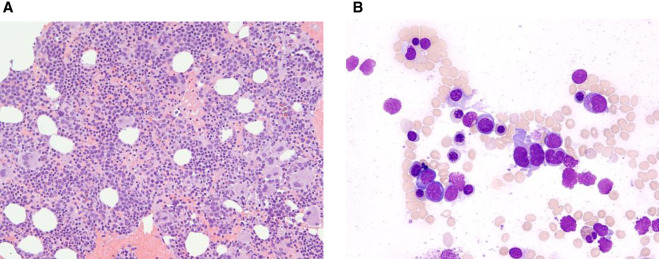
The bone marrow at diagnosis was high normocellular with a prominent increase in erythropoiesis with dyserythropoiesis, increased megakaryopoiesis with dysplasia including hypolobated megakaryocytes and nuclear lobe separation, and increased blasts. (*A*) Bone marrow clot section, hemotoxylin and eosin (H&E), 200×. (*B*) Bone marrow aspiration, Wright–Giemsa stain, 500×.

She was evaluated by pediatric gastroenterology and diagnosed with Gilbert's syndrome with a homozygous *UGT1A1* variant. Labs at this visit were remarkable for 3% peripheral blasts, and she was directed to the emergency department for admission to the Hematology/Oncology service. A bone marrow exam revealed MDS with excess blasts (MDS-EB2, 14% blasts) with flow cytometry showing increased blasts positive for CD34, HLA-DR, and partially positive for CD117, CD38, CD13, and CD33 (Fig. 1). These bone marrow features are less common in patients with *SAMD9/SAMD9L* germline predisposition, who more commonly present with refractory cytopenia of childhood (Sahoo et al. 2021). Fluorescence in situ hybridization (FISH) was performed on the bone marrow sample using our institutional MDS panel and results are notable for a deletion of 7q or monosomy 7. Karyotype from the bone marrow was positive for inversion 3 and monosomy 7 and next generation sequencing revealed *KRAS, MPL, KMT2C*, and *SF3B1* (subclonal) variants. An inherited bone marrow failure panel was sent from peripheral blood and revealed a novel *SAMD9* variant (heterozygous c.4460A > G; p.Lys1487Arg) and two heterozygous variants of uncertain significance, *TET2* and *SBDS* (Table 1). Skin biopsy was performed and confirmed *SAMD9* as a germline variant for this patient. Family history was significant for iron deficiency in her mother, a paternal first cousin who underwent SCT for hemophagocytic lymphohistiocytosis, maternal grandmother with thyroid cancer, and maternal great grandmother with breast cancer.

**Table 1.** Variant table

Gene	Chromosome location	HGVS DNA reference	HGVS protein reference	Variant type	Predicted effect	dbSNP/dbVar ID	Genotype	Parent of origin	Observed effect (if shown to be different from predicted effect)
*SAMD9*	7q21.2	c.4460A > G	p.Lys1487Arg	Variation of uncertain significance	Missense	rs556478940	Heterozygous	Maternal	MutationTaster, REVEL, SIFT, PolyPhen, benign
*SBDS*	7q11.21	c.139C > T	p.Leu47Phe	Variation of uncertain significance	Missense	N/A	Heterozygous	Paternal	REVEL, deleterious (0.84); MutationTaster, Deleterious)
*TET2*	4q24	c.3472G > A	p.Ala1158Thr	Variation of uncertain significance	Missense	N/A	Heterozygous	Maternal	MutationTaster, deleterious; REVEL, 0.39

### Diagnostic Confirmation and Treatment

Our patient received two cycles of azacitidine. She tolerated these cycles well, with minimal side effects of nausea and vomiting. A bone marrow evaluation after her two cycles of azacitidine showed 10%–12% blasts based on CD34 immunostain performed due to hypospicular bone marrow aspirate smear. The other morphologic findings were essentially unchanged. This treatment plan prevented her disease from progressing to AML and bridged her to stem cell transplant as the next phase of therapy.

**Figure 2.**
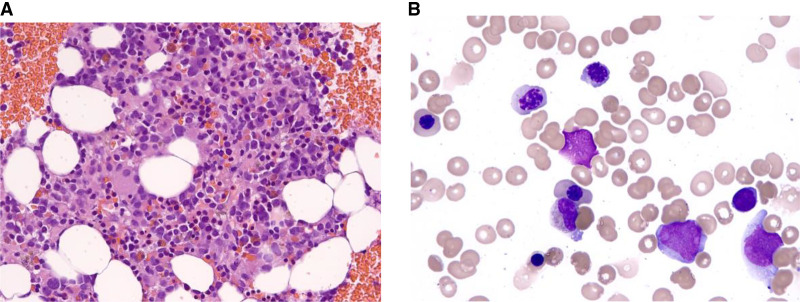
The bone marrow at the time of recurrence was variably cellular with increased erythropoiesis with dyserythropoiesis, decreased granulopoiesis, and increased megakaryopoiesis with dysplasia. Blasts constituted 17% of the differential count. (*A*) Bone marrow clot section, hematoxylin and eosin (H&E), 400×. (*B*) Bone marrow aspiration, Wright–Giemsa stain, 1000×.

She received a haploidentical bone marrow stem cell transplant from her father after fludarabine, thiotepa, and cyclophosphamide conditioning regimen (Hyder et al. 2019). She did not receive a busulfan-based conditioning regimen based on the increased risk of mortality seen in patients with Gilbert's disease and opted for a non-total-body irradiation–based regimen because of the concern for hepatotoxicity in the setting of Gilbert's disease (McDonald et al. 2016). She received post-transplant cyclophosphamide, mycophenolate mofetil, and sirolimus as graft versus host disease (GVHD) prophylaxis. She engrafted on Day +31 with 100% donor chimerism in whole blood and peripheral CD3+ cells, as well as in the bone marrow. Her post-transplant course was complicated by acute GVHD of the skin (max grading: stage 3 skin; overall grade 2), chronic sinusitis, and BK viremia. She was found to have relapsed MDS on Day +49 with detection of minimal residual disease (MRD) by flow cytometry at 0.04% on screening bone marrow exam. A rapid taper of immune suppression was performed. Her disease did not respond to discontinuing her immune suppression and she had worsening of acute skin GVHD. A bone marrow on Day +114 showed variably cellularity with 17% blasts, and cytogenetics performed on this bone marrow sample inversion 3 and monosomy 7, MRD was 28% (Fig. 2). She was subsequently started on venetoclax and azacitidine with a plan to perform a second transplant once her disease was under control. Unfortunately, her disease progressed rapidly after one cycle of venetoclax and azacitidine, and she ultimately succumbed to relapsed disease (Fig. 2).

## DISCUSSION

This case report describes the finding of a germline *SAMD9* variant (c.4460A > G) in a patient with a new diagnosis of MDS with excess blasts without underlying MIRAGE syndrome. This variant has not been reported in the literature as disease-associated and may signify an aggressive form of MDS. Other *SAMD9* pathogenic variants have been associated with MIRAGE syndrome, bone marrow failure, a predisposition to monosomy 7 MDS, and AML. SAMD9 is commonly deleted within a 7q21 cluster in patients with myeloid neoplasia. Mice with *SAMD9L* haploinsufficiency were shown to develop myeloid malignancies mimicking human disease with monosomy 7 (Nagamachi et al. 2013).

Familial testing was done on our patient confirming inheritance of the *SAMD9* (c.4460A > G) variant from her mother. Additionally, two siblings share the *SAMD9* variant, whereas the father as well as a younger sibling do not have this variant. Marrow evaluation of the two siblings with the variant have been done with one sibling showing hypocellular marrow with decreased granulopoiesis and normal blast count. The other sibling's marrow was within normal limits. Germline *SAMD9* variants have variable clinical courses (Bluteau et al. 2018). This is evident as Schwartz et al. (2017b) describe a family noted to have an inherited *SAMD9* variant resulting in three children developing MDS with monosomy 7 at an early age, whereas the mother remained unaffected. Of the three siblings, the two eldest had a successful bone marrow transplant from a matched unrelated donor and the youngest sibling is monitored annually as they remain asymptomatic (Schwartz et al. 2017b). Our patient's mother and siblings are unaffected, demonstrating the variable clinical course of germline *SAMD9* variants. The variation in the clinical course indicates the importance of monitoring the siblings as they are younger and could potentially develop MDS later. It has also been shown in our patient and in the literature that some patients with *SAMD9* MDS are at increased risk of relapse (Davidsson et al. 2018). The clinical heterogeneity with *SAMD9* variants is suggested by the lack of symptoms in the patient's mother and two siblings. Based on prior literature and current knowledge of the antiproliferative nature of gain of function *SAMD9* variant, the highly proliferative nature of this patient's disease could certainly be secondary to the acquired activating variants noted of *KRAS*, *MPL*, and *SF3B1* (Wong et al. 2018). It may be clinically valuable to know and understand the specific variant in *SAMD9* as this could help provide prognostic information that could impact treatment of the patient.

Our patient presented with a new diagnosis of MDS, shortly after a COVID-19 infection. It has been suggested that there may be a link between COVID-19 infections and new hematologic malignancy diagnosis. Case reports have found various hematologic malignancies occurring either simultaneously or shortly after a diagnosis of COVID-19, raising concern for a probable association between the infamous virus and malignancies (Khan et al. 2020; Nekooghadam et al. 2021; Costa et al. 2022). Although, this raises the question of whether the COVID infection may have triggered the development of MDS in the setting of an underlying genetic predisposition, it is unlikely a COVID-specific effect but rather that any virus can induce an interferon response that could lead to a SAMD9-related marrow failure given this patient's underlying germline variant.

It is not clear from our one case report how this variant *SAMD9* variant impacts a diagnosis of MDS with excess blasts but warrants further analysis in patients who have a refractory and significant disease course. *SAMD9* genetic testing for patients with MDS could help identify family members who would require routine screening for the various clinical presentations of either MIRAGE syndrome or MDS, as well as consideration of fertility preservation of that patient. If patients can be predicted to have a worse prognosis or aggressive disease, the treatment plan could be modified and potentially improve outcomes. It is therefore imperative to further investigate this novel variant to aid in the future care of patients with this *SAMD9* variant.

## METHODS

Our participant was identified through clinical presentation to our inpatient unit. After the diagnosis of MDS was made, peripheral blood was sent to Prevention Genetics for Inherited Bone Marrow Failure testing and bone marrow aspirate was sent to Hematologics for Foundation One testing. Fluorescence in situ hybridization (FISH) studies were performed internally at Indiana University. Targeted testing of known proband variants was performed by Prevention to identify additional family members with these variants, for donor status considerations and appropriate clinical care of family members. Further chart review was conducted after obtaining verbal consent from the patient's mother, who is next of kin as the patient has since passed away. A review of the literature using PubMed identified previous literature that was used to develop our discussion.

## ADDITIONAL INFORMATION

### Data Deposition and Access

This variant was submitted to ClinVar (https://www.ncbi.nlm.nih.gov/clinvar/) and can be found under accession number VCV001727220. Patient consent was not granted to deposit raw sequencing data.

### Ethics Statement

Verbal consent was obtained by patient's mother (next of kin) as patient had passed away at the time of writing the case report. This was obtained over the phone with a two-physician consent process. Per our institutional standard, case reports are not considered human subject research and therefore do not need IRB approval.

### Author Contributions

All authors contributed equally in the conceptualization of the case report, drafting the initial manuscript, and in critical review of the manuscript. All authors were involved in the clinical care of the patient's case outlined in the case report.

### Funding

No funding was obtained for this case report.

### Competing Interest Statement

The authors have declared no competing interest.

### Referees

Craig M. Forester

Anonymous
